# Evaluation of TB/HIV Collaborative Activities: The Case of South Tongu District, Ghana

**DOI:** 10.1155/2020/4587179

**Published:** 2020-05-22

**Authors:** Vasco Ayere Avoka, Eric Osei

**Affiliations:** ^1^Department of Epidemiology and Biostatistics, School of Public Health, University of Health and Allied Sciences, Ho, Ghana; ^2^Department of Population and Behavioural Sciences, School of Public Health, University of Health and Allied Sciences, Ho, Ghana

## Abstract

**Background:**

There is a complex interaction between infection with human immunodeficiency virus (HIV) and tuberculosis (TB) infection that results in a synergistic increase in their prevalence, morbidity, and mortality. In Ghana, 32% of TB cases were estimated to be coinfected with the human immunodeficiency virus and acquired immune deficiency syndrome (HIV/AIDS) epidemic HIV, with the highest number of coinfections in the Volta Region. This study assessed the extent of linkage between the TB and HIV collaborative activities in the South Tongu District of Ghana.

**Method:**

The study employed both qualitative and quantitative methods to assess the coverage of activities to reduce the burden of TB in people living with HIV and the coverage of activities to reduce the burden of HIV in TB patients and explored the barriers to collaborative activities from the providers' perspective.

**Results:**

The study showed that 344 (94.8%) HIV-positive clients were screened for TB, of which 10 (8.5%) were bacteriologically confirmed. Among those positive for TB, 6 (60%) received cotrimoxazole preventive therapy (CPT) and antiretroviral therapy. Sixty-seven (93.1%) TB patients were screened for HIV. Of these, 28 (38.9%) were retropositive, among whom 14 (50%) received anti-TB treatment. However, there were no records of isoniazid preventive therapy (IPT) for these patients. Inadequately trained personnel leading to work overload, manual record-keeping, lack of staff motivation, and absence of “enablers” packages for patients were identified as barriers to TB/HIV collaboration.

**Conclusion:**

Overall, there was a moderate linkage between TB and HIV collaborative activities in the study setting. Notwithstanding, there exist some barriers that mitigate against the successful implementation of the collaborative process from the providers' perspective, hence we recommend for measures to ensure effective, efficient, and sustained integrated TB/HIV activities by addressing these barriers.

## 1. Background

There is a complex interaction between infection with human immunodeficiency virus (HIV) and tuberculosis (TB) infection that results in a synergistic increase in their prevalence, morbidity, and mortality [1]. The coexistence of TB and HIV is a great public health problem looming as a potential pandemic in Ghana and other African countries [2]. In recognition of the dual burden and impact of these two diseases, the World Health Organization (WHO) in 2004 initiated the TB/HIV collaborative activities as an integral part of national and international responses to the joint TB/HIV treatment at all levels of service delivery [1]. As part of the policy, TB patients are to be screened for HIV and positive cases are provided with cotrimoxazole preventive therapy (CPT) and antiretroviral therapy (ART) where indicated. HIV patients are also to be regularly screened for TB and managed appropriately [2].

Despite the proven efficacy of TB/HIV treatment and prevention, studies in Ghana have found that the prevalence of HIV in TB patients is approximately 25-30% and that as many as 50% of patients with a chronic cough could be HIV positive [2]. Cultural and system-wide differences between HIV and TB care providers present operational difficulties for providing effective and appropriate interventions which have contributed to a lack of progress in expanding collaborative TB/HIV activities [3].

Since the national launch of TB/HIV collaborative activities in Ghana in 2007 and operation in 2009, not many studies have focused on the operational effectiveness of the integrative process, especially in the Volta Region. This study, therefore, assessed the TB/HIV collaborative activities in a district hospital in the Volta Region of Ghana.

## 2. Materials and Methods

### 2.1. Study Setting

South Tongu is one of the 26 administrative districts of the Volta Region. The population of South Tongu District, according to the 2010 Population and Housing Census, is 87,950 representing 4.1% of the region's total population. Males constitute 45.5% and females represent 54.5%. About eighty-seven (87.1%) of the population resides in rural localities with a sex ratio (number of males to 100 females) of 83.5%.

### 2.2. Study Design and Population

The study employed both qualitative and quantitative methods to assess the coverage of activities to reduce the burden of TB in PLHIV and the coverage of activities to reduce the burden of HIV in TB patients and explored the barriers to collaborative activities from the providers' point of view. The quantitative tool was used to measure the linkage of the TB and HIV collaborative activities. Data were extracted from the TB and HIV registers at the Voluntary Counselling and Testing (VCT) unit and Chest Clinic from 1st January 2014 to 31st December 2016 in the district hospital. All cases of TB and HIV registered at the hospitals during the period were included for the analysis. The qualitative method allowed for the identification of barriers to collaboration from the healthcare providers' perspective. The TB and HIV coordinators from the district hospital were interviewed to establish the barriers to collaboration.

### 2.3. Data Analysis

Quantitative data were exported to IBM SPSS for analysis. Data cleaning and validation were done to ensure data quality before analysis was carried out. Descriptive statistics such as proportions, frequencies, mean, and standard deviation were used to describe the population. Qualitative data were analysed thematically, and pattern building was done using a priori themes that were used to design the study as well as emergent ones. Interviews were recorded directly in words using pen and paper. Data was then organized manually into easily retrievable sections assigned with unique codes, or by breaking up field notes into sections identified by date, or by context using both sentences and paragraphs as the units of analysis. These codes (salient themes, recurring ideas, patterns of beliefs, etc.) were then refined or combined to form themes or categories of issues.

### 2.4. Ethical Issues

This study was approved by the Ghana Health Service Ethical Review Committee with reference no. GHS-ERC 57/05/17. Participation was completely voluntary, and participants could choose not to participate or leave the study at any time during data collection.

## 3. Results

### 3.1. Characteristics of HIV and TB Cases

There were 363 HIV cases registered during the study period. Of these, females accounted for nearly three-quarters (272, 74.7%). The majority (215, 59.2%) belong to the economically active age group (20-39 years). The minimum and maximum ages were 12 and 77 years, respectively, with the median age of 36 years (Table 1).

Of the 72 TB cases registered during the study period, the majority 37 (51.4%) were males. The majority (57, 79.2%) were in the age group 20-59 years. The minimum and maximum ages were 3 and 87 years, respectively, with a median age of 40 years (iqr = 18). Forty-three (59.7%) of them were pulmonary positive and 1 (1.4%) was an extrapulmonary case (Table 2).

### 3.2. TB and HIV Collaborative Activities

**Figure 1:**
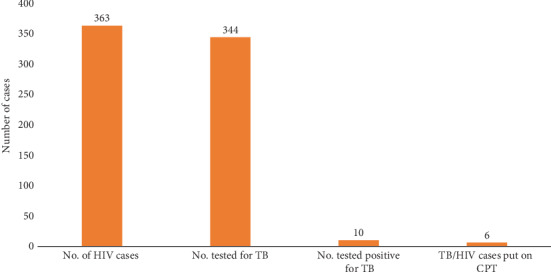
TB collaborative activities among HIV patients.

Of the 363 HIV cases, 344 (94.8%) of them were screened for TB, of whom 10 (8.5%) were bacteriologically confirmed TB; six (60%) of the HIV-positive TB patients had access to cotrimoxazole preventive therapy (CPT) and antiretroviral therapy (Figure 1).

**Figure 2:**
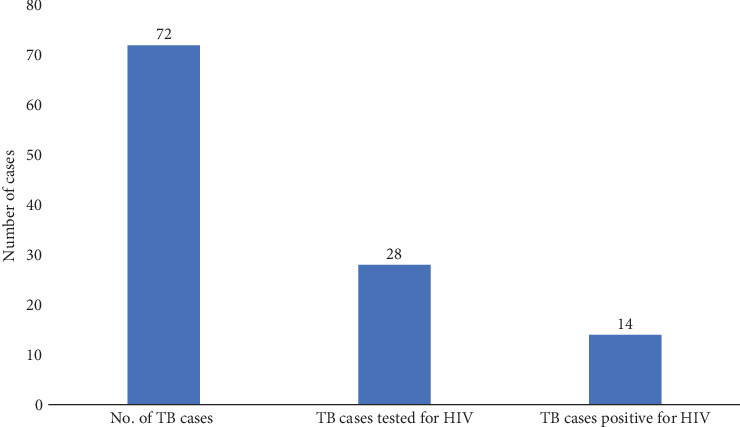
HIV collaborative activities among TB patients.

Among the 72 TB cases, 67 (93.1%) were screened for HIV, of whom 28 (38.9%) were positive for HIV. Of the 28 HIV-positive TB patients, 14 (50%) were put on antituberculosis treatment (Figure 2). There were no records of isoniazid preventive therapy (IPT) for TB/HIV patients.

### 3.3. Barriers to Collaboration

Several barriers that mitigate against the successful implementation of TB and HIV control collaborative activities were enumerated by key health personnel involved in TB and HIV control at the hospital. Prominent among the barriers mentioned were inadequate staff leading to work overload. This was what the TB facility coordinators had to say:

“*I am the only qualified staff here (the DOTS centre) so I am not able to go out to the field (to follow up cases) and leave my patients in the ward. This has affected service delivery because I am not able to trace defaulters.*” (facility TB coordinator)

The Voluntary Counselling and Testing unit was located on a different block of the hospital and was being overseen by a senior nurse. When the in-charge was asked about the challenges of the unit, she also reiterated the lack of qualified personnel.

“*We are three in this unit but the other two are not regular staff because of that the workload on me is too much hence I am not able to visit some clients because the clinic runs every day and I have to be here at all times. This makes me very tired so am not able to work effectively.*” (facility VCT coordinator)

Manual data capture and records keeping was also mentioned as one of the barriers to successful TB/HIV collaborative activities at the hospital. At the DOTS centre, patients' information was recorded in folders and papers and stored in a cupboard. According to the nurse-in-charge, the process makes retrieval of folders and some records very cumbersome and time wasting.

“*Because the records are kept manually, it is difficult to retrieve some folders sometimes when they (patients) come for follow up visits. Some of the folders can get missing or destroyed when they are packed together. It is also difficult to monitor to see whether a referred patient to VCT unit go to the unit or not because the records are kept in folders which cannot be seen and used by more than one person at a time.*” (facility TB coordinator)

To ensure that TB/HIV clients adhere to their treatment regimens, food was added as a package which as described by service providers as an “enablers” package. However, this package was scrapped in 2015 after funds dwindled, which posed a major challenge to the TB/HIV collaborative activities.

“*The enabling package should be re-introduced so that patients would adhere to their treatment since this serves as a motivation for them. Most patients do not visit the clinic as scheduled and when they come and if we ask them, they say they the drugs make them a week to travel to the clinic*.” (TB coordinator)

As confirmed by Mbilinyi et al. [4], health worker motivation plays a critical role in the quality of service rendered by healthcare providers. The lack of motivation for staff working in TB/HIV units adversely affect the quality of the service rendered according to the hospital DOTS centre in-charge.

“*The lack of motivation may not affect me as a person but other staff like the OPD nurses, or the laboratory personnel. If they are not given some motivation, they would not help us with the detection of cases. This can affect our case detection rate.*” (facility TB coordinator)

## 4. Discussion

TB screening among people living with HIV almost reached universal in this study site, similar to what Lima et al. [5] found in their study in Johannesburg, South Africa, where more than 9 in 10 TB patients received an HIV test. The high screening rates as observed in this study mean that the healthcare providers adhere to the WHO recommendation that all TB patients must be screened for HIV. This implies that treatment outcomes in TB patients would be improved and also transmission to contact persons would be reduced. Early detection of TB among people living with HIV, followed by prompt initiation of treatment, can improve patient treatment outcomes and reduce transmission in communities and healthcare settings [6]. TB screening is also essential in evaluating PLHIV infection or AIDS for isoniazid preventive therapy (IPT) eligibility in order to avoid the risk of isoniazid monotherapy resistance [6]. WHO emphasizes the relevance of screening for TB in PLHIV to reduce the impact of TB among people living with HIV (PLHIV).

In order to lessen the burden of TB in HIV patients, new HIV-positive TB patients should receive a TB regimen containing 6 months of rifampicin and should be put on ART regardless of CD4 count as soon as possible within the first 8 weeks of antituberculosis treatment [3]. In this study, 6 in 10 of all eligible HIV-positive TB patients received TB treatment. This implies that the other 4 cases go untreated for TB, and they would continue to infect others from the healthy population who they come into contact with.

Studies have proven that IPT is as efficacious but safer than rifampicin- and pyrazinamide-containing regimens used for the prevention of latent TB infection and effective in reducing the incidence of TB and death from TB in HIV-infected patients with a positive tuberculin skin test result [3]. Although WHO recommend IPT as part of the standard care for HIV patients without active TB, IPT uptake could not be measured in this study as it was not indicated in the registers whether patients receive IPT or not. This study result is similar to a study in Ethiopia which found that IPT intake could not be measured due to an incomplete TB register [7]. While this study could not measure the level of IPT uptake, in Ethiopia, it was found that 19.4% of TB and HIV/AIDS patients received IPT [8]. IPT has been proven to reduce the incidence of TB cases among HIV patients. A study in Ethiopia to compare the incidence rate of TB and TB-free survival time and identify factors associated with the development of TB among HIV-infected individuals on pre-ART follow-up found that IPT use was associated with a 50% reduction in new cases of tuberculosis, and implementing the widespread use of IPT had the potential to reduce TB rates substantially among HIV-infected individuals in addition to other tuberculosis prevention and control efforts in resource-limited settings [9].

In this study, more than 9 in 10 TB patients during the period were screened for HIV, similar to what was found in an earlier study in Ghana by [10]. This result favourably compares with a prospective observational study to determine the burden and treatment of HIV in tuberculosis patients in Papua Province, Indonesia, which established that 85.2% of all TB patients were tested for HIV [11]. A quantitative assessment at 3 clinics in Johannesburg, South Africa, to evaluate TB/HIV integration at the primary care level established that the proportion of clients newly diagnosed with HIV who were screened for TB symptoms could not be determined, because it was not systematically documented [12]. Correspondingly, Rie et al. found that in Ethiopia 85% of TB patients at DOTS clinics were tested for HIV/AIDS [13]. Our result is not in conformity with [12, 13] where only 75% of TB patients had their HIV status documented and only a little more than half of TB patients were tested for HIV, respectively.

Cotrimoxazole preventive therapy (CPT) is composed of multiple antimicrobial agents that prevent a range of secondary bacterial and parasitic infections in eligible TB adults and children living with HIV and should be implemented as an integral component of the HIV chronic care package [3]. In terms of access to CPT, this study found that only half of all HIV-positive TB patients registered during the period received CPT, similar to what was found in Uganda [14]. However, the CPT treatment rate in this current study is lower than what was found in Ethiopia where 8 in 10 HIV-positive TB patients received CPT [11, 14] and in India where more than 9 in 10 HIV-positive TB patients were given CPT [7]. The rest of the eligible HIV-positive TB patients who did not receive CPT would be more prone to secondary and parasitic infections, and this may affect their treatment outcomes [7].

HIV is a predictor for active TB both in people with new infection or with latent *Mycobacterium tuberculosis* infection. Improving access to TB prevention, treatment, care, and support services for health-care workers, as well as of workers in congregate settings, is, therefore, crucial [3].

Antiretroviral therapy (ART) greatly improves the survival and the quality of life of TB patients living with HIV and prevents HIV transmission and reduces mortality risk between 54% and 95% [3]. In this study, of the 72 TB cases registered, ART service could not be established because there was no documented record on ART in the TB register. Pontororing et al. found that in South Africa ART coverage among patients with TB could not be ascertained as ART was not documented in the TB register or on the TB treatment card [12]. The results of this study, however, contradicts what was found in Cameroon where 82% of TB/HIV coinfected patients were enrolled on ART [15, 16]; the authors found that 52% of the HIV-positive TB patients in their study had access to ART during TB treatment. The inability of this study to measure ART utilization is because HIV-positive TB clients are usually referred to the VCT unit for such services which do not report progress back to the DOTS centre. It is therefore proper that ART for eligible patients be given at the DOTS centre or the VCT staff gives feedback to the DOTS centre staff.

Many studies have shown that there are challenges to the successful implementation of collaborative activities of TB/HIV and this study is not different. Focal persons for the two programmes who were interviewed in this study stated the lack of adequate personnel, leading to high workload as a major challenge to successful TB/HIV collaboration. In sub-Saharan Africa, many patients were attended to by a few providers at the DOTS centres [16]. Retirement from active service by service providers could account for the lack of adequate health personnel in this study. It is likely that some facility personnel after working for some time go for further studies while the elderly ones retire from active service when they attain 60 years.

Lack of staff motivation is one of the challenges mitigating against the successful implementation of TB/HIV activities [17].

Findings from the previous study in Ghana also cited poorly motivated staff as a major barrier to the TB/HIV collaboration [10]. Motivation and better remunerations can boost the morale of health staff to deliver to their fullest especially for those who work with communicable and infectious diseases like TB and HIV. For instance, a study to establish the effect of motivation on the performance of employees at GT Bank Ghana found that bonuses motivate staff to perform at work and motivation was seen as a silent contributing factor to increased performance [18], similar to Jasmi who found that motivated employees not only influence their work performance but also the whole organization's performance and business productivity [19].

Manual record-keeping was also found to be a challenge to the TB/HIV collaborative activities in the facility. Due to the nature of manual records, it was not easily accessed by more than one person at a time and usually difficult to transport [20].

The inability to be accessed by more than one person at a time makes it impossible or difficult for staff at the unit to monitor clients who have been referred to the other unit for other services. Again, navigation through manual records is cumbersome and highly time-consuming and this may delay the delivery of health services, supported by [21] who concluded that experts and policymakers believe that electronic health records are beneficial to the patient and society if they are used in a meaningful way.

“*The termination of ‘enabling' packages as a motivation and support found to be a challenge to the successful collaboration of TB/HIV collaborative activities. Due to the burden of the TB pills, the program initiated a package in the form of food to motivate and help them adhere to and complete their treatment in time. However, this ‘enabling' package of late has been irregular in supply to the TB patients.*” (TB coordinator)

This may lead to high default rates and nonadherence and also affect negatively TB patients' participation in HIV services, as evidenced by Ciobanu et al. in 2014 who observed that provision of enablers such as cash and food to TB patients significantly improves the treatment success rate and should be continued [21]. Also, lack of food was among the other factors identified as a barrier to adherence to TB treatment in patients on concomitant TB and HIV treatment.

## 5. Conclusion

Though some aspects of the collaborative activities are commendable, more effort is needed to ensure the linkage of all activities between TB and HIV/AIDS programmes and to address the exiting factors that mitigate against the implementation of these collaborative activities to reduce the burden of HIV on TB patients and vice visa. If more pragmatic measures are not put in place, the 90-90-90 target cannot be achieved.

## Figures and Tables

**Table 1:** Age and sex distribution of HIV cases registered (2014-2016).

Period	2014 (*n* = 134)		2015 (*n* = 112)		2016 (*n* = 117)		All years (*n* = 363)	
Variable	*n*	%	*n*	%	*n*	%	*n*	%
Sex								
Female	103	76.9	82	73.2	87	74.4	272	74.7
Male	32	23.1	30	26.8	30	25.6	92	25.3
Age range (years)								
≤19	4	3.0	1	0.9	3	2.6	8	2.2
20-39	78	58.2	65	58.0	72	61.5	215	59.2
40-59	43	32.1	42	37.5	37	31.6	122	33.6
≥60	9	6.7	4	3.6	5	4.3	18	5.0

**Table 2:** Characteristics of tuberculosis cases registered from 2014 to 2016.

Variable	2014		2015		2016		All years	
	*n* = 26	%	*n* = 23	%	*n* = 23	%	*n* = 72	%
Sex								
Female	16	61.5	12	52.2	7	34.4	35	48.6
Male	10	38.5	11	47.8	16	69.6	37	51.4
Age (years)								
≤19	1	3.9	0	0.00	1	4.6	2	2.8
20-59	18	69.2	22	95.6	17	73.9	57	79.2
≥60	7	26.9	1	4.4	5	21.7	13	18.0
Disease type								
PP	14	53.9	15	65.2	14	60.9	43	59.7
PN	12	46.1	8	34.8	8	34.8	28	38.9
EP	0	00.0	0	00.0	1	4.4	1	1.4
Treatment outcome								
Completed	9	34.62	4	18.2	4	17.4	17	23.9
Cured	12	46.15	13	59.1	13	56.5	38	53.5
Died	5	19.23	5	22.7	4	17.4	14	19.7
LTF	0	0.00	0	00.0	2	8.7	2	2.8

PP: pulmonary positive; PN: pulmonary negative; EP: extrapulmonary; LTF: lost to follow-up.

## Data Availability

The datasets used and/or analysed during the current study are available from the corresponding author on reasonable request.

## Abbreviation

AIDS: Acquired immune deficiency syndrome
ART: Antiretroviral therapy
CHPS: Community-based health planning and services
CPT: Cotrimoxazole prophylaxis therapy
DOTS: Directly observed therapy short course
GHS: Ghana Health Service
HIV: Human immunodeficiency virus
IPT: Isoniazid preventive therapy
IRIS: Idiopathic immune reconstitution syndrome
NACP: National AIDS Control Program
NTP: National Tuberculosis Program
PLHIV: People living with human immunodeficiency virus
SSA: South-Saharan Africa
TB: Tuberculosis
VRHD: Volta Regional Health Directorate
VCT: Voluntary counselling and testing
WHO: World Health Organization
